# Physicochemical parameters and modes of interaction associated with the micelle formation of a mixture of tetradecyltrimethylammonium bromide and cefixime trihydrate: effects of hydrotropes and temperature

**DOI:** 10.1039/d3ra04748b

**Published:** 2023-10-17

**Authors:** Md Al Amin Hossain, Tamanna Islam, Javed Masood Khan, Md. Tuhinur R. Joy, Shamim Mahbub, Salman A. Khan, Anis Ahmad, Mohammad Majibur Rahman, Md. Anamul Hoque, Shariff E. Kabir

**Affiliations:** a Department of Chemistry, Jashore University of Science and Technology Jashore 7408 Bangladesh joytuhinur@yahoo.com; b Department of Food Science and Nutrition, College of Food and Agricultural Sciences, King Saud University Riyadh 11451 Saudi Arabia; c Nuclear Safety, Security & Safeguards Division, Bangladesh Atomic Energy Regulatory Authority Agargaon Dhaka 1207 Bangladesh; d Physical Sciences Section (Chemistry), School of Sciences, Maulana Azad National Urdu University Hyderabad 500032 Telangana India; e Sylvester Comprehensive Cancer Center, University of Miami, Miller School of Medicine Miami FL USA; f Department of Environmental Sciences, Jahangirnagar University Savar Dhaka 1342 Bangladesh; g Department of Chemistry, Jahangirnagar University Savar Dhaka 1342 Bangladesh ahoque_ju@juniv.edu; h Department of Chemistry, Jagannath University Dhaka 1100 Bangladesh

## Abstract

The interaction between an antibiotic drug (cefixime trihydrate (CMT)) and a cationic surfactant (tetradecyltrimethylammonium bromide (TTAB)) was examined in the presence of both ionic and non-ionic hydrotropes (HTs) over the temperature range of 300.55 to 320.55 K. The values of the critical micelle concentration (CMC) of the TTAB + CMT mixture were experienced to have dwindled with an enhancement of the concentrations of resorcinol (ReSC), sodium benzoate (NaBz), sodium salicylate (NaS), while for the same system, a monotonically augmentation of CMC was observed in aq. 4-aminobenzoic acid (PABA) solution. A gradual increase in CMC, as a function of temperature, was also observed. The values of the degree of counterion binding (*β*) for the TTAB + CMT mixture were experienced to be influenced by the concentrations of ReSC/NaBz/NaS/PABA and a change in temperature. The micellization process of TTAB + CMT was observed to be spontaneous (negative standard Gibbs free energy change (Δ*G*^0^_m_)) at all conditions studied. Also, the values of standard enthalpy change (Δ*H*^0^_m_) and entropy change (Δ*S*^0^_m_) were found negative and positive, respectively (with a few exceptions), for the test cases indicating an exothermic and enthalpy–entropy directed micellization process. The recommended interaction forces between the components in the micellar system are electrostatic and hydrophobic interactions. In this study, the values of Δ*C*^0^_m_ were negative in aqueous NaBz, ReSC, and PABA media, and positive in case of NaS. An excellent compensation scenario between the enthalpy and entropy for the CMT + TTAB mixed system in the investigated HTs solutions is well defined in the current work.

## Abstract 2

TTAB surfactant and CMT drug interaction in presence of NaBz.

## Graphical abstract

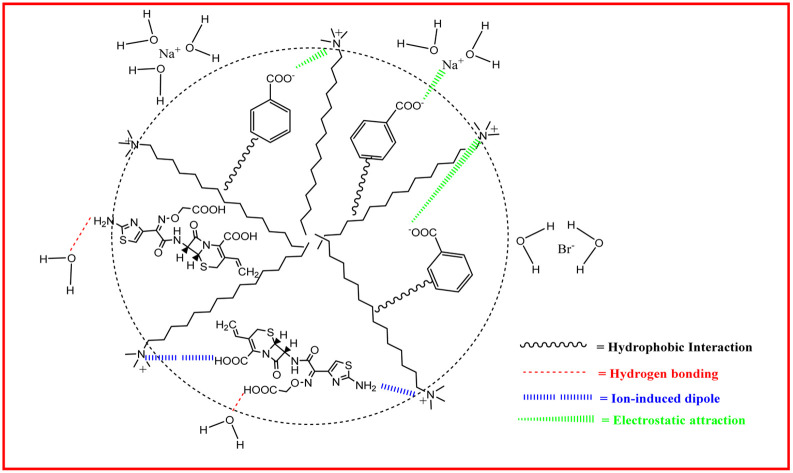

## 1. Introduction

A prominent group of chemicals, surfactants, possess a polar hydrophilic unit and an apolar hydrophobic unit in each molecule and are receptive to a broad variety of chemical responses.^1^ There is extensive use of these supramolecular systems in many different kinds of research fields and a broad array of uses in many industrial applications (most notably, in foods, fluid emulsion, beauty products, detergents, medicines, controlled medication delivery, extraction of oil, nano- and biotechnology domains, including catalysis, and cosmetics industries). The most remarkable property of surfactants is their ability to form micelle at a certain concentration, which is defined as the critical micelle concentration (CMC).^2–4^ Surfactant molecules can interact with the biologically active molecules in drug formulation and drug delivery,^5–7^ proteins,^8,9^ biomedical substances,^10^ biopolymers,^11–13^ amino acids.^14–16^ Again, the solubility of hydrophobic substances, like additives, drugs, dyes, and stabilizer, dramatically increases due to the intrinsic characteristic of surfactant to form micelle. In fact, surfactants' self-aggregate nature in solution, lowering surface tension and increasing the solubility of poorly soluble compounds, are well documented.^7^ Consequently, the functionality of active pharmaceutical ingredients (API) relies heavily on the nature of surfactant micelles because they can transport and solubilize API species in the micelle core or hydrophobic cavity of the micelle, where the micelles act as nano-containers for the drug delivery system or transportation of drug to the active site of human or animal body. The solubilization of API happens at and above the CMC of surfactant, which enhances the dissolution of API. The molecules of API experience a higher entry rate into the bloodstream after solubilization. However, excess use of surfactant (exceeding CMC at specified conditions) leads to lower absorption of API by lowering the API's chemical potential.^17^ As solubilization starts at CMC and maximum absorption of drug is attained at the CMC, precise determination of CMC is essential to get the maximum activity of drugs. In addition, the introduction of hydrotropes can change the accumulation and metabolic activity of micellar complexes, specifically their emulsification capacities and to provide an extra impact on drug formulation.^18–24^ Hence, the primary purpose of the mixed micelle of surfactant in presence of different additives is to aid in the enhancement of solubility of the drug and take a crucial part in the formulation and delivery of that drug.

The term “hydrotrope” (HT) was first introduced by Neuberg in 1916, which refers to an organic molecule that has both hydrophobic and hydrophilic characteristics, like surfactants, and has the ability to increase the dissolution, by the formation of micelle, of certain other organic materials or minerals in aqueous or aquatic solution of salt.^25,26^ The presence of a hydrotropic molecule can boost its solubility by creating a weak van der Waals contact with a less water-soluble molecule through an appealing dipole–dipole attraction or a weak van der Waals force.^27^ In the current investigation, we have carried out an analysis of the impact of four HTs- (sodium benzoate (NaBz), sodium salicylate (NaS), *p*-aminobenzoic acid (PABA) and resorcinol (ReSC)) on the cationic surfactant tetradecyltrimethylammonium bromide (TTAB) by the conductometric measurement technique. These additives have profound biological and real-life applications. Among these chemical compounds, NaBz, and NaS are the salts of benzoic acid, which are frequently used to preserve foods and fruit juices, in addition, to serving as vital ingredients in the creation of resin, dyes, plasticizers, inks, and medicinal goods.^28,29^ PABA is widely available in nature and utilized in the biomedical industry,^30^ the PABA's UV-absorbing qualities have led to its widespread application in sunscreen compositions. Its transformation into the specialized azo dyes and crosslinking agents are some of its further applications, in addition, PABA is also employed as a biodegradable insecticide. To improve color durability and shield hair from UV damage, certain hair coloring products contain PABA derivatives. The chemical compound resorcinol (ReSC), with formula C_6_H_4_(OH)_2_, is the 1,3-isomer (or *meta*-isomer) of benzenediols. Insoluble in chloroform and carbon disulfide, ReSC forms crystals from benzene as colorless needles, which are easily soluble in alcohol, water, and ether.^31^ In the prescribed treatments, it is present in greater concentrations and is, usually, found in topical acne treatments, which is sold as 2% or less concentration.^32^ Furthermore, it is also employed to treat hidradenitis suppurativa, however, there is little evidence that it can speed up the healing of the lesions.^33^ In order to optimize the drug formulation and alter the action of that medicine, it is vital to investigate the mode of utilization of additives in this surfactant–drug system.

A key consideration when developing novel pharmaceutical formulations is the analysis of drug–surfactant interactions. It is possible to evaluate the mechanistic insights and interactions between a medicine and a surfactant using a variety of inquiry techniques by finding out some physicochemical parameters. Also, in most industrial products surfactants have significant uses to achieve the desired stability, activity functions, *etc.* In these cases, the interactions between solutes (polymer/gels/organic compounds, *etc.*) and surfactants occurred which significantly modified the aggregation character of the surfactants.^34–39^ Measurement of conductivity,^34–36^ surface tension,^36–38^ light scattering techniques,^35,37,38^ steady-state fluorescence spectroscopy,^36,37^ simple and differential UV/visible spectroscopic studies,^39^ and small-angle neutron scattering (SANS)^40^ methods were used to evaluate the physicochemical features of the aggregation process of the solutes–surfactant system. In the literature, several studies have revealed the mode of interaction of surfactants and HTs, emphasizing the widespread applications in antibacterial medicines.^18–23,40–47^ The technological and physiological benefits of mixed micelles, instead of individual surfactant systems, have shown ample scientific, commercial, and pharmacological utilizations and alternative surfactants and HTs mixtures can be developed for drug administration.^40^ The surfactant's chemical composition, as well as the gel/surfactant ratio, kind of solvent, and test temperature, all had an impact on the interaction's strength and nature.^48^ It was further demonstrated that the solution characteristics of the drug (ibuprofen)–surfactant mixes (sodium octyl sulfosuccinate), as well as the interactions between the drug and the surfactant were affected by the presence of both simple and hydrotropic electrolyte (aggregation parameters, interfacial properties, and thermodynamics of aggregate formation).^43^ Alfaifi *et al.*^41^ investigated the interactions of hydrotropes *ortho*-toluidine hydrochloride (*o*-TDH) and *para*-toluidine hydrochloride (*p*-TDH) with phenothiazine drug promethazine hydrochloride (PMH) at different temperatures.

Islam *et al.*^42^ also revealed their report that the ceftriaxone sodium drug had the hydrophilic interaction with surfactant TX-100 in the presence of NaS, but for NaBz, this interaction was found insignificant. However, at various temperatures, Rehman *et al.*^34^ discovered the presence of electrostatic interactions in the association process of polylactic acid (PLA) with ionic surfactants-hexadecyltrimethylammonium bromide (CTAB) and sodium dodecyl sulphate (SDS), whereas Hanif *et al.*^39^ reported benzothiophene (BZT) partitioning in micellar medium was aided by both electrostatic and hydrophobic factors. Rub *et al.*^44^ reported that H-bonding and electrostatic contacts are the predominant forces amid TX-100 and MNH in the occurrence of PABA, although hydrophobic interactions are thought to be the dominant force among the component species used in aq. NaBz and NaS media. Paul *et al.*^45^ investigated an in-depth mechanistic approach to the hydrotropic solubilization of drug molecules, which is applicable in the pharmaceutical industries. With the unending importance of HTs, especially in the pharmaceutical industry, some future directions for this technique are worth mentioning.

**Scheme 1:**
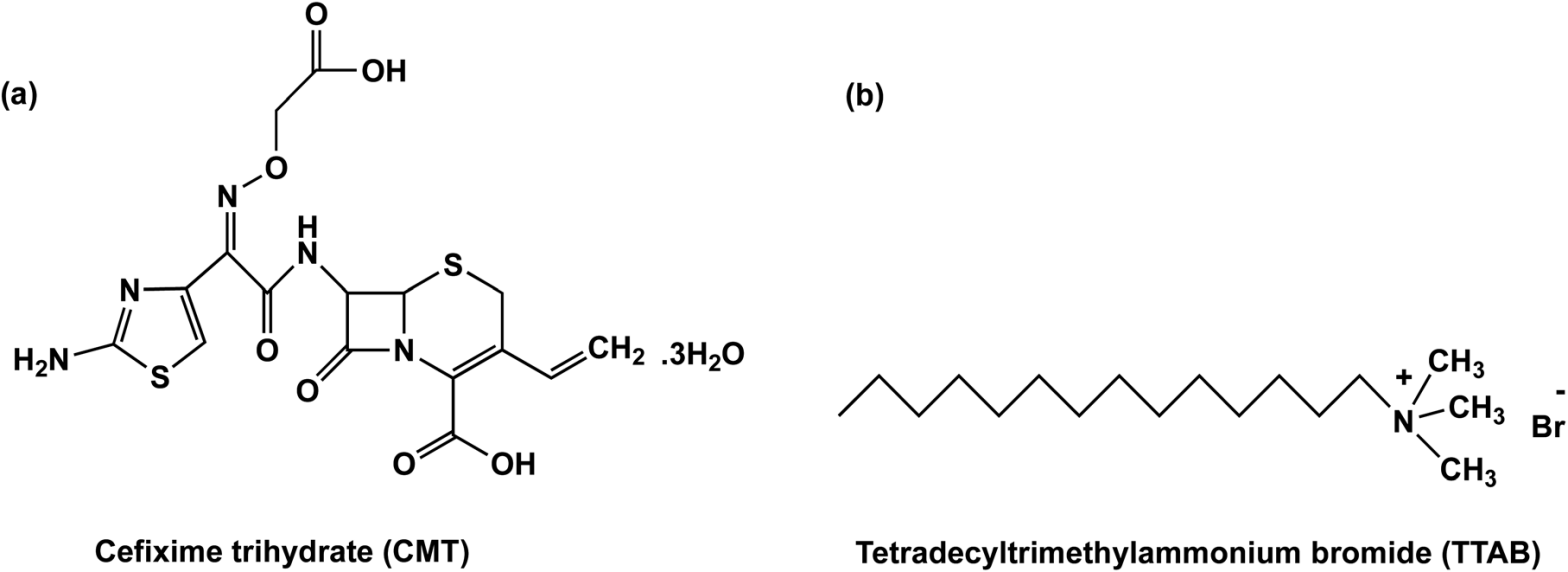
Chemical structure of (a) CMT, (b) TTAB.

TTAB (Scheme 1), is a cationic surfactant and an organic building block.^49,50^ It creates hemimicelles and functions as a surface-active aid in the interfacial separation of anionic species produced from acids. In this study, we have used the third-generation antibiotic drug (CMT) (Scheme 1), which is a wide-ranging cephalosporin antibacterial drug, made semi-synthetically from coastal fungi. Similar to penicillin, the beta-lactam antibacterial cefixime prevents the development of cell walls in bacteria by preventing the synthesis of peptidoglycans. It is used to treat infections of urinary tract, throat infections, pneumonia, gonorrhea, and bronchitis. In addition, the drug is a good remedy for skin and soft tissue infections; able to stop group A and B beta-hemolytic streptococci.^51^ CMT, generally, has a negative charge and is attracted to micelles' positive head groups. Understanding the effects of this interaction on drug–surfactant associations is essential because when a drug is administered, it should interact with the electrolyte molecules effectively.

Currently, there are rare study reports in the literature on the investigation of the interaction of CMT and TTAB in aqueous solutions of HTs. The goal of the current research is to develop the formulation of CMT in micellar solution to get its maximum activity and also to monitor the impact of HTs on the micellization characteristics of cationic surfactant using the conductometric method in aqueous solutions at five distinct temperatures. The physicochemical parameters, like CMC, *β*, degree of ionization (*α*), and thermodynamic properties (standard free energy change (Δ*G*^0^_m_), standard entropy change (Δ*S*^0^_m_), and standard enthalpy change (Δ*H*^0^_m_), molar heat capacity (Δ*C*^0^_m_)), and enthalpy–entropy compensation variables were also calculated to examine the interaction between CMT and TTAB. The main purpose of the mixed system is to aid in the enhancement of the solubility of drug. The employed drug is found to be completely soluble within the used concentration in surfactant media. However, structurally it contains both hydrophilic and hydrophobic parts in the structure, but it could not behave like other amphipathic substances (does not form aggregates). Therefore, herein we studied the effect of ionic and non-ionic hydrotropes (HTs) on interaction of antibiotic drug CMT with TTAB surfactant and also deliberate the effect of temperature variation on their interaction. We believe the findings will be useful in adopting cationic surfactants for drug formulation, drug delivery and in enhancing drug penetration to avoid the side effects of drugs on the human body. Also, another important application of worth mentioning will be the use of HTs as additives in drug formulation in the pharmaceutical industries.

## 2. Experimental

### 2.1. Materials

In this investigation, chemicals of analytical grade have been used. CMT (99% purity, CAS: 125110-14-7) was utilized; it was collected from Gonoshasthaya Pharmaceuticals Ltd Bangladesh. TTAB (purity 99%, CAS: 1119-97-7, source: Sigma-Aldrich (USA)), NaBz (purity 98%, CAS: 532-32-1, source: Merck, India), NaS (purity 98%, CAS: 54-21-7, source: Scharlau Chemicals, Spain), PABA (purity 98%, CAS: 150-13-0, source: Sigma-Aldrich (USA)), and ReSC (purity 98%, CAS: 108-46-3, source: Sigma-Aldrich (USA)) were used in the present study. For the preparation of the solution at the experimental temperatures (300.55–320.55 K), distilled-deionized H_2_O was employed, which has a specific conductivity (*κ*) below 1.8 μS cm^−1^.

### 2.2. Method

A digital conductivity meter (Jenway 4510, UK) having ±0.5% precision was used to determine the specific conductivity (*κ*) of the experimental mixture in the applied solvents by following the procedures outlined in the literature.^52–56^ The KCl (0.01 M) solution was employed for calibrating the conductivity meter. In this study, at first, TTAB solutions (50 mmol kg^−1^) were made in a mixture of CMT, HT, and water, with the concentrations of CMT being held constant and HT concentration varied in a specific range to investigate the concentration effect on the micellization. After placing 25 mL of the solvent (CMT + HT + H_2_O medium preserving the selected CMT and HT concentration) in a test tube, the solution's *κ* values were determined. The test tube was immersed in a water thermostat bath (RM6 Lauda) with outstanding thermal accuracy (±0.2 K) during the research to preserve a unique thermal state. After that, the TTAB solution (50 mmol kg^−1^) was progressively added with a set volumetric interval to the 25 mL solvent. The *κ* readings were measured using a conductivity meter for each experimental run after sufficient mixing and approval of enough time to accomplish the temperature equilibration. This whole set of work was performed in two ways; (a) by changing temperature at a constant concentration of additive, and (b) by changing concentration of additive at a constant temperature. Then, the *κ* values *versus* TTAB concentration (*C*_TTAB_) plot has then been used to evaluate the CMC by using Origin software. The additional calculations were carried out in MS Excel software.

## 3. Results and discussion

### 3.1. Assessment of CMC, *α*, and *β* in the case of association of the TTAB + CMT mixture in HT media

**Fig. 1:**
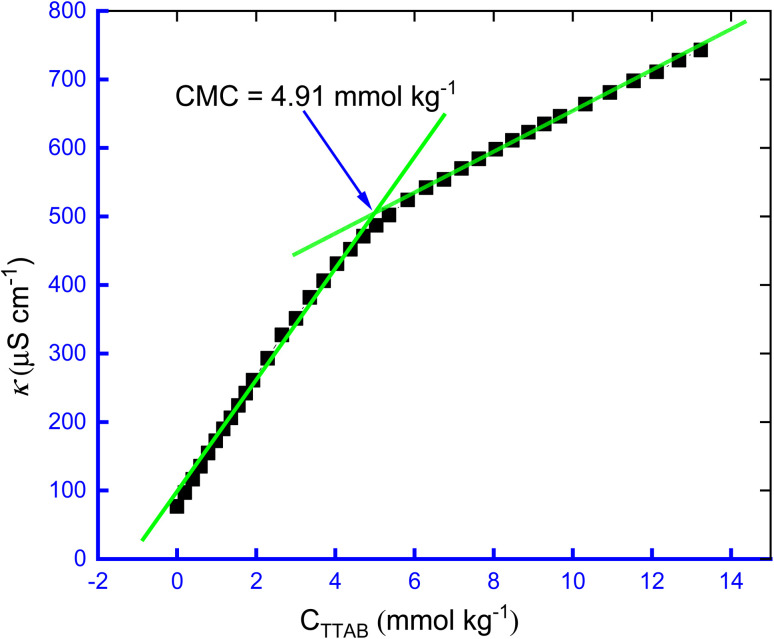
The plot of *κ* against *C*_TTAB_ for the TTAB + CMT (0.3 mmol kg^−1^) mixture in 0.5 mmol kg^−1^ NaBz solution at 300.55 K.

The aggregation processes of the TTAB + CMT mixture were carried out in the existence of a hydrotropic aqueous solution. The CMT drug is insoluble in water, but the inclusion of HTs in the water made the CMT soluble. However, TTAB is strongly soluble in water and generates TTA^+^ and Br^−^ ions in the solution. These dissociated ions cause an increase in the conductivity of solutions, which makes a straight line in the *κ vs. C*_TTAB_ plot. However, after the addition of certain concentration of a specific surfactant, the increasing rate of conductivity with concentration of surfactant becomes slower because surfactant molecules start to generate aggregated molecules (micelle) which act as a single polymeric molecule, *i.e.*, the rate in increasing of specific conductivity of the solution drops and the slopes of the straight line after micellar point flattens out as the micellar ions cannot move as rapidly as the molecular ions can, and create a breakpoint in the plot as seen in Fig. 1. Two factors are responsible for the relatively gradual decrease in the increasing rate of specific conductivity that occurred following the development of CMC compared to the values seen in the pre-CMC state: an initiation of the TTAB micelle (a) and the establishment of the Helmholtz double layer of electrical charge, which is brought on by the abundance of liberated Br^−^ ions in the surfactants micellar phases (b). Another consequence is the fact that the neutralization of the TTAB surface charge stabilizes the surfactant micelle simultaneously, which lowers the electrostatic interactions of repulsion.^57^

The specific concentration in the breakpoint is the concentration at which the surfactant molecules tend to aggregate to form a supramolecular structure, called micelle. The initial macromolecule or micelle forming concentration is called CMC.^57–60^ The degree of ionization (*α*) and counter ion binding (*β*) are so crucial parameters to investigate the micellization process of surfactant and these parameters are closely related to CMC, all these three parameters have been evaluated by the conductometric method in this study. The change in conductivity at the micelle formation state is more clearly noticeable than other methods, furthermore, this method is more reliable and easier to handle than surface tension measurement or spectroscopic method. In Fig. 1, a typical plot of *κ vs. C*_TTAB_ has been displayed.

To assess the *α* for the creation of micelle of TTAB + CMT system, the ratios of the slope of the pre-micellar straight-line to the slope of the post-micellar straight-line are employed, *i.e.*, the values of *α* is estimated by the *S*_2_/*S*_1_ ratio, *i.e.*, *α* = *S*_2_/*S*_1,_ where *S*_1_ and *S*_2_ are the corresponding slopes of the pre-micellar and the post-micellar straight lines, respectively.^52^ The repulsive forces (electrostatic) among the surfactant head groups increase the magnitude of *α*, requiring a larger amphiphilic concentration for micellization to occur. Surfactant is employed as a catalyst, because adding it to the reaction medium enhances its surface area.^57^ It is acknowledged that lower *α* values result in quicker surfactant micellization and a higher opportunity of micelles forming at lower concentrations of surfactant. Consequently, the value of *α* was subtracted from one to determine *β* for the aggregation of TTAB + CMT mixture, *i.e.*, *β* = 1 − *α*. The value of CMC of 4.91 mmol kg^−1^ for the TTAB + 0.3 mmol kg^−1^ CMT mixture in 0.5 mmol kg^−1^ NaBz solution at 300.55 K was observed in this study. The reported CMC values for TTAB, found in water at various operating temperatures, generally, vary from 3.74 to 4.39 mmol kg^−1^.^58^ It is important to note that utilizing the conductivity technique, Banjare *et al.*^59^ experienced a value of CMC of TTAB surfactant in H_2_O of 3.80 mmol L^−1^ at 298.15 K. Therefore, HTs have a significant effect on the micellization of TTAB as in the presence of HTs, the CMC value is different from the reported values of pure TTAB in aq. media (Tables 1 and 2).

**Table 1:** Values of CMC and *β* for the aggregation of TTAB + CMT (0.3 mmol kg^−1^) mixture in HT solutions with a studied range of concentrations at 310.55 K^a^

Medium	*c*_HTs_ (mmol kg^−1^)	CMC (mmol kg^−1^)	*α*	*β*
NaBz + H_2_O	0.05	5.45	0.51	0.49
	0.10	5.25	0.46	0.54
	0.50	5.18	0.42	0.58
	1.00	4.95	0.40	0.60
	3.00	6.50	0.48	0.52
	5.00	6.86	0.51	0.49
NaS + H_2_O	0.05	5.41	0.57	0.43
	0.10	5.21	0.55	0.45
	0.50	5.10	0.53	0.47
	1.00	4.58	0.56	0.54
	3.00	7.37	0.66	0.34
	5.00	8.81	0.80	0.20
ReSC + H_2_O	1.00	4.82	0.39	0.61
	5.00	4.29	0.37	0.63
	10.00	3.90	0.35	0.65
	15.00	4.06	0.37	0.63
	20.00	4.12	0.40	0.60
	25.00	4.32	0.41	0.59
PABA + H_2_O	1.00	4.38	0.30	0.70
	5.00	4.70	0.32	0.68
	10.00	4.90	0.35	0.65
	15.00	5.07	0.36	0.64
	20.00	5.11	0.38	0.62
	25.00	5.30	0.40	0.60

a Standard deviation: CMC = 5%, *α* = 7%, and *β* = 7%.

**Table 2:** Values of CMC and *β* for the aggregation of TTAB in hydrotropic solutions of certain concentration at the specific temperature range^a^

Medium	*C*_HTs_ (mmol kg^−1^)	*T* (K)	CMC (mmol kg^−1^)	*β*
NaBz + H_2_O	0.50	300.55	4.91	0.64
		305.55	5.08	0.60
		310.55	5.18	0.58
		315.55	5.38	0.44
		320.55	5.61	0.40
NaS + H_2_O	0.50	300.55	4.45	0.50
		305.55	4.88	0.49
		310.55	5.10	0.47
		315.55	5.31	0.45
		320.55	5.54	0.40
ReSC + H_2_O	10.00	300.55	3.65	0.69
		305.55	3.82	0.68
		310.55	4.00	0.65
		315.55	4.25	0.64
		320.55	4.63	0.62
PABA + H_2_O	10.00	300.55	4.26	0.68
		305.55	4.43	0.66
		310.55	4.65	0.65
		315.55	4.97	0.64
		320.55	5.20	0.62

a Standard deviation: CMC = 5%, and *β* = 7%.

### 3.2. Effect of HTs on the TTAB + CMT micellization

**Fig. 2:**
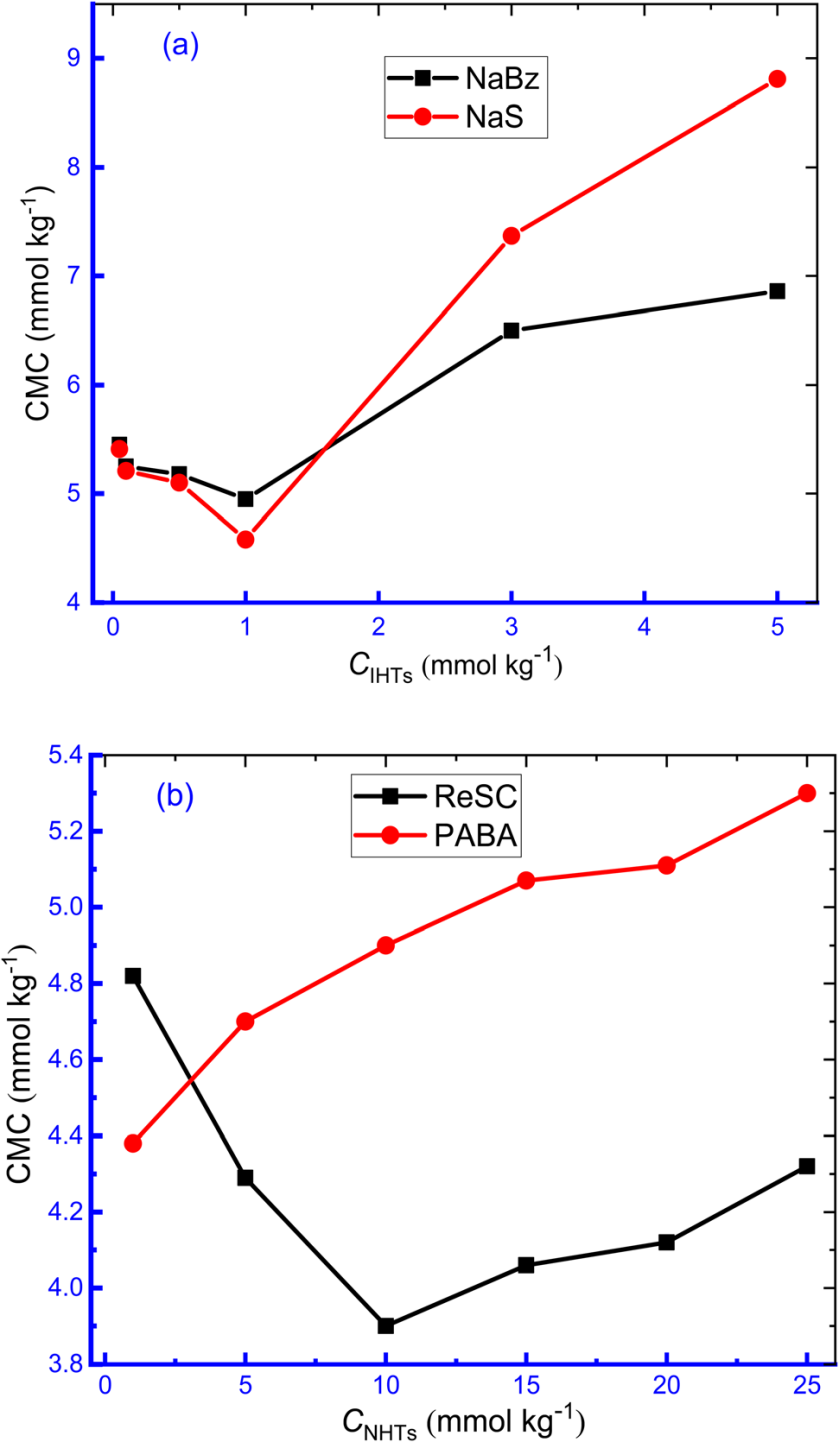
The changes of CMC with the augmentation of concentration of HTs for the micellization of TTAB + 0.3 mmol kg^−1^ CMT mixture at 310.55 K (a) IHTs, (b) NHTs.

It is vital to investigate the utilization of additives in this surfactant–drug system since these additives have profound biological and real-life uses. To study the impact of the organic compounds (HTs) on the aggregation of TTAB + CMT mixture, aqueous solutions of HTs, *e.g.*, NaBz, NaS, ReSC, and PABA with different concentrations, were taken as the study media. NaBz and NaS are IHTs and ReSC and PABA are NHTs. The selected range of concentration of ionic HT (IHT) was 0.05–5.00 mmol kg^−1^ and for non-ionic HT (NHT), the corresponding value was in the range of 1.00–25.00 mmol kg^−1^. Such concentration range of HTs in the study of micellization of cationic surfactants including TTAB was chosen randomly just to examine the impact of HTs on association of TTAB + CMT mixture. The additives, used is the study, may have some degree of amphiphilic character due to their molecular structures. These, typically, do not show surface active properties in the same way that dedicated surfactant molecules can perform. Although NaBz shows moderate surface activity^61^ and decreases surface tension,^62^ NaS can enhance the viscosity of aqueous surfactant solutions.^63^ Surface activity of ReSC is relatively low compared to traditional surfactants and PABA is not known to exhibit surface activity but is used as an intermediate in the synthesis of certain pharmaceuticals and sunscreens. The addition of anionic HTs leads to the reduction of the surface tension of TTAB and enhances the surface activity.^64^ The compensation of surface charge of ionic surfactants, in the presence of oppositely charged HTs, causes deeper packing in the aggregation and can behave like non-ionic surfactants, which causes accumulation of monomers at the surface.^65^ The CMC and *β* values for the micellization of the working system at 310.55 K in HTs media are represented in Table 1. The variations of CMC with an increase in HT concentration are shown in Fig. 2.

With the increase in concentration of IHTs (NaBz, NaS), the values of CMC declined slowly (NaBz: 5.45 mmol kg^−1^ to 4.95 mmol kg^−1^, NaS: 5.41 mmol kg^−1^ to 4.58 mmol kg^−1^) by rising concentration up to 1.00 mmol kg^−1^ and then, started to upsurge rapidly with the increase in concentration of HTs (Table 1 and Fig. 2). This finding expresses that the formation of micelle is more favourable at 1.00 mmol kg^−1^ and less favourable in other studied concentrations. The higher concentration of NaBz and NaS inhibits micelle formation, which results in the augmentation of CMC, as published in the literature.^66^

The initial reduction of CMC values for the CMT + TTAB mixture with the increase of NaS, NaBz HTs concentration at 310.15 K could be attributed to the effective charge neutralization at the micellar surface, *i.e.*, reduction in repulsion amongst TTA^+^ ions by SS^−^/Bz^−^ ions, which results in earlier micelle formation and reduction in CMC.^64^ The strongly anionic character of the chosen IHTs causes them to bind strongly to oppositely charged surfactant ions when added to TTAB + CMT solutions, which reduces the effectiveness of the head groups area due to the screening effect. This resulted in the development of geometric change from cylindrical to spherical micelles caused by the cationic HTs, implying a strong attractive connection between the two.^67^ Because of this, the formation of micelles occurs at significantly lower concentrations than in pure water, with the reduction in CMC being influenced by the concentration of HTs.^68^ A large number of counter ions bind to ionic micelles, and the counterions are primarily bound to micellar surfaces by the electrical fields created by the head groups, as well as by the specific interactions among the head group of ions and the counter ion.^64^ The abnormal rise in the CMC of TTAB at higher HT concentrations (higher than 1.00 mmol kg^−1^) appears to be caused by the electrostatic action of ionic HTs and their hydrotropic activity, which results in an increase in solubilizing capacity and a decrease in solvent polarity.^69^ The high solubilizing capacity stabilizes the surfactant monomers by delaying the formation of micelles and the consequences in the result of enhancing CMC values.

The CMC values of TTAB + CMT mixture in the appearance of PABA showed an increasing trend with increasing concentration of PABA; while in aqueous ReSC solution, the CMC values gradually decreased with rising the concentration to a minimum value and, then, increased again with the increase in concentration. These findings indicate that lower ReSC concentration facilitates the aggregation process and higher concentration functions oppositely. PABA, a nonionic HT, inhibits to micelles formation of TTAB + CMT mixed system that results in an upward trend of CMC value with the rise in concentration. Polymer-based surfactant system (CTAB/PEO system) also experienced an upsurge of micellar concentration with an augmentation of the composition of the polymer.^35^ Hanif and co-workers^39^ described the upsurge of CMC for the micellization of SDS with the introduction of benzothiophene (BZT). They illustrated the water structure breaking impact of BZT by disrupting H-bond of water and thereby the micellization of SDS becomes delayed.^39^ There is a possibility of generation of positively charged nitrogen atom in the resonance structure of PABA, which repels the head group of TTAB and inhibits micellization. Therefore, there is an increase in the values of CMC in the manifestation of PABA. The variation of dielectric constant (DC) in different concentrations of solvent has an effect on the upward trend of the CMC value, hydrophobic nature is another culprit for this trend.^70,71^ The introduction of organic solvents results in the reduction of DC of water, which enhances the repulsive force between the hydrophilic head group of surface-active agents, consequently, the aggregation of surfactant molecules is delayed resulting in higher CMC value.^72–75^ Another theory proposed that the solubility of the hydrophobic part of surfactant gets an upsurge due to the reduction of the cohesive force of water molecules in the inclusion of aqueous organic solvents, like HTs. Hence, the interactions between non-polar part of the surfactant and the organic solvent prevent the formation of micelle, *i.e.*, higher concentration of surfactant is required to form micelle. The *β* values for TTAB + CMT mixture were found to be dependent on HTs concentration, *i.e.*, *β* values experienced to be increased followed by a reduction with the increase of the concentration of ReSC, NaBz, NaS, while a monotonical reduction was observed for PABA. The reduction of *β* values for the micellization of cationic surfactant, with the increase of polymer concentration, was also reported in the literature.^36^ The binding of counterion at the micelle surface reduces the interhead groups' repulsion and, thus, the micellization becomes feasible. The higher counterion binding causes more favorable conditions for the aggregation of surfactant molecules and, thus, an early micellization happens. Our estimated results are well agreement with this phenomenon.

### 3.3. Effect of temperature on the CMC and *β* of TTAB + CMT micellization in hydrotropic media

The temperature is a significant experimental condition on which the physicochemical properties, along with the thermodynamics of a system, mostly rely and this parameter serves the critical role in the investigation of the mode and nature of interactions between surfactant and drug. The values of CMC and *β* for the TTAB + CMT mixture, at different temperatures in the addition of HTs (NaBz, NaS, ReSC, and PABA), are shown in Table 2.

**Fig. 3:**
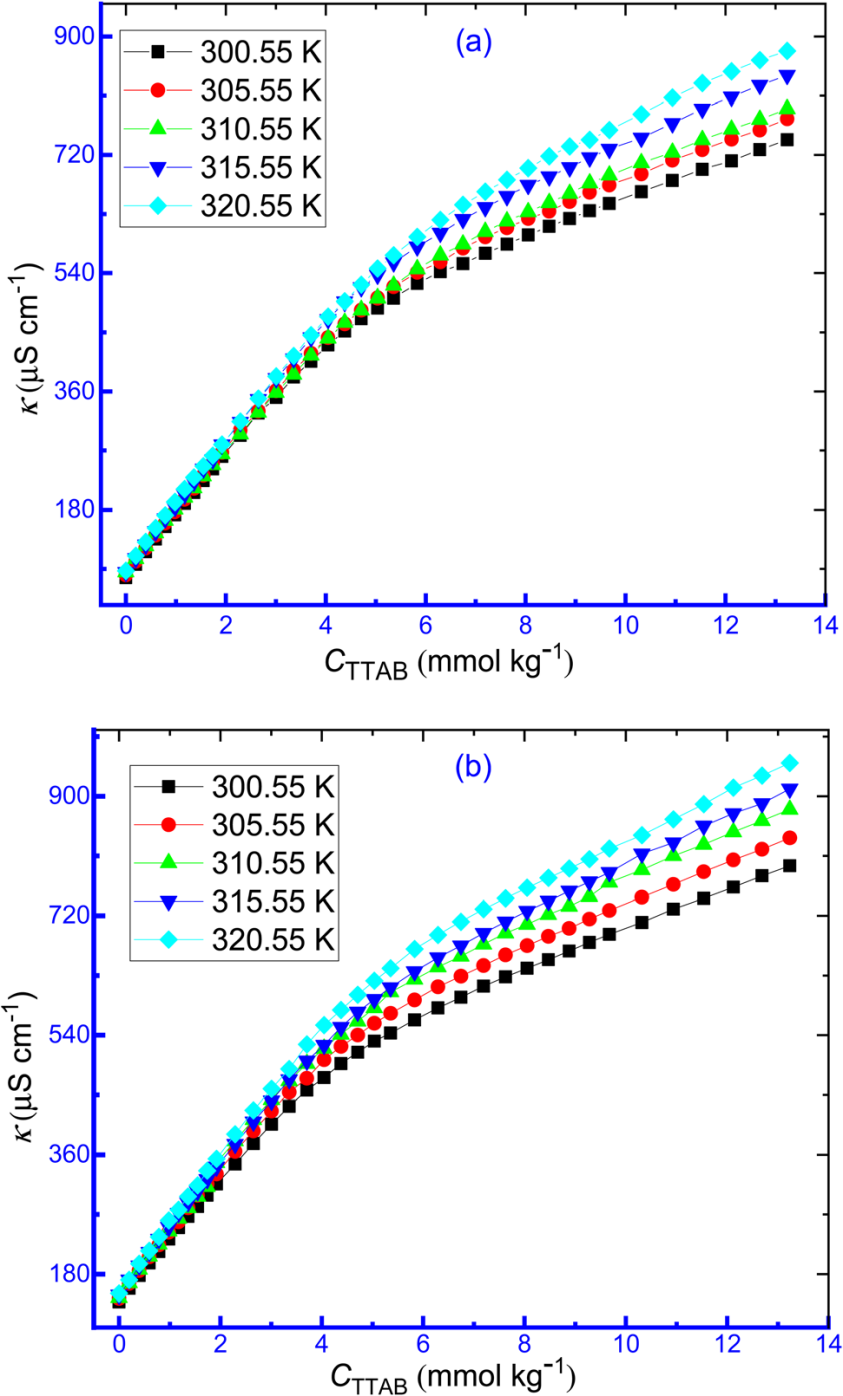
The plot of *κ versus C*_TTAB_ for the TTAB + CMT mixture having (a) aqueous solution of NaBz (0.5 mmol kg^−1^) and (b) aqueous solution of PABA (10 mmol kg^−1^) at different temperatures.

**Fig. 4:**
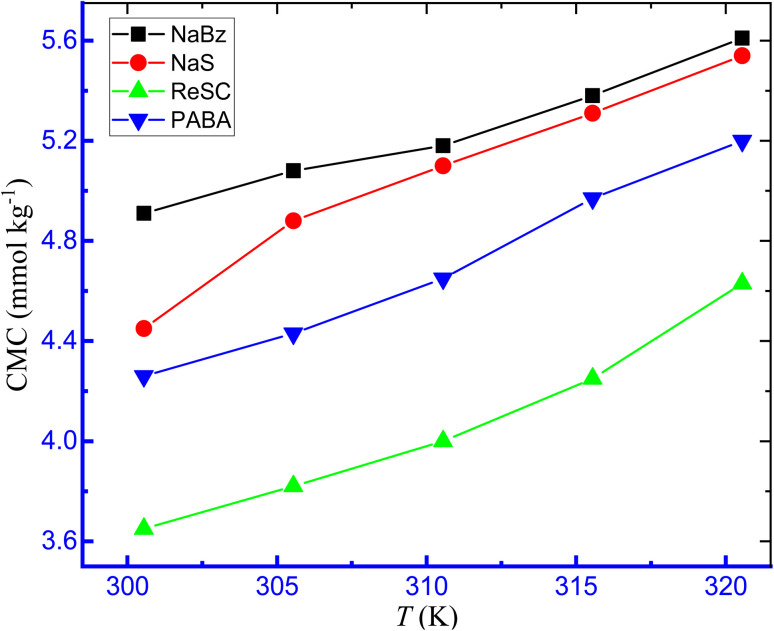
Plots of CMC *versus T* for the aggregation of TTAB and CMT mixture in aq. hydrotropic media.

With rising temperatures, it was discovered that the *κ* of TTAB + CMT mixture in attendance of IHT (NaBz) and NHT (PABA) increased as the mobility of the ions increased with temperature (Fig. 3). Fig. 4 shows the opposite relationship between CMC and temperature for the association of TTAB + CMT mixture in an aqueous hydrotropic medium. For the TTAB + CMT mixed systems in HT solutions, with rising temperature, it was observed that the CMC values increased gradually.

The temperature has a paramount effect on the aggregation of any surfactants or drug–surfactant mixture and can alter the micellization environment to change the CMC value or micellar size or shape. In the present study, higher temperature made the system in a disfavorable environment to form micelle, *i.e.*, micelle formed at a greater concentration of surfactants in the presence of both IHTs and NHTs. The CMC values of the TTAB + CMT mixed system in the introduction of NaBz, NaS, ReSC, and PABA showed an upward trend with the rising of temperature of the system. The order in the values of CMC in the existence of these mediums is: CMC_NaBz_ > CMC_NaS_ > CMC_PABA_ > CMC_ReSC_. By changing the water structure, or the kind of hydrations surrounding the monomeric surfactant molecules and CMT-modified TTAB micelle with rising temperature, it is possible to demonstrate how temperature affects the CMC values. The surfactant molecules experience both forms of hydration (hydrophilic or hydrophobic) at their monomeric form, but the aggregated TTAB system is thought to be affected by the hydrophobic hydration. For TTAB or TTAB + CMT mixed solutions, it is anticipated that both hydrophilic and hydrophobic hydration will decrease with the rising of temperature.^76^ As previously stated, as the temperature rises, a decreasing hydrophilic hydration encourages the production of micelles, whereas a decreasing hydrophobic hydration disfavors the micellization. For these results, there are two chief issues that interfere with the construction of micelle. One of them is the de-solvation (a drop in hydrophilic hydration) of the head group of the amphiphile molecules (TTAB), which encourages the development of micelle and, therefore, causes the CMC to decline. Another significant element that hinders the formation of micelles is the decrease of water arrangement near the non-polar part of TTABs and drugs, *i.e.*, the value of CMC undergoes an upsurge.^77^ Therefore, whether the values of CMC grow or reduce at the examined range of temperature depends on the extent of the aforementioned two parameters. In the case of the TTAB + CMT mixed system in an aqueous medium, as well as in the presence of HTs, the last factor controlled the process of micellization more significantly than the first one within the range of temperature. Hence, the CMC values of TTAB + CMT mixture in the presence of HTs increase as the temperature rises. Again, the increase of temperature causes higher molecular or ionic motion, and, thus, the counterion binding experiences a reduction with the augmentation of temperature. Similar results were also observed in our earlier study.^76,77^ Rehman *et al.*^36^ also reported the increasing trend of CMC for pure surfactants (SDS and CTAB) or surfactants with polylactic acid (PLA) solution. It was also reported that the micellization of 3-(1-alkyl-3-imidazolio) propane-sulfonate [ImS3-R] was easier at lower temperatures but became disfavoreable at higher temperatures.^38^ Therefore, the ionic head groups experience higher inter-head group repulsion, and the micellization is delayed.

### 3.4. Thermodynamics of the micellization of TTAB + CMT mixture in HTs media

The thermodynamic parameters are crucial in illuminating the process of surfactant micelle production and, ultimately, the energetically spontaneous process of surfactant aggregation (TTAB). In the attendance of aqueous solution of HTs at the studied temperature range, the thermodynamic quantities of the examined surfactant's micellization were calculated. The following equation was employed to estimate the standard Gibbs free energy change (Δ*G*^0^_m_) of association of surfactant.^58,78–80^

1 Δ*G*^0^_m_ = (1 + *β*)*RT* ln *X*_CMC_

Here, the mole fraction of CMC is denoted by *X*_CMC_. *R* and *T* stand for the molar gas constant and temperature in kelvin, respectively.

The standard enthalpy change (Δ*H*^0^_m_) required to generate a micelle was obtained by applying the formula shown below:^79^

2 Δ*H*^0^_m_ = −(1 + *β*)*RT*^2^(∂ln *X*_CMC_)/∂*T*

The temperature dependent nature of ln *X*_CMC_ can be described by the eqn (3):

3 ln *X*_CMC_ = *A* + *BT* + *CT*^2^

**Fig. 5:**
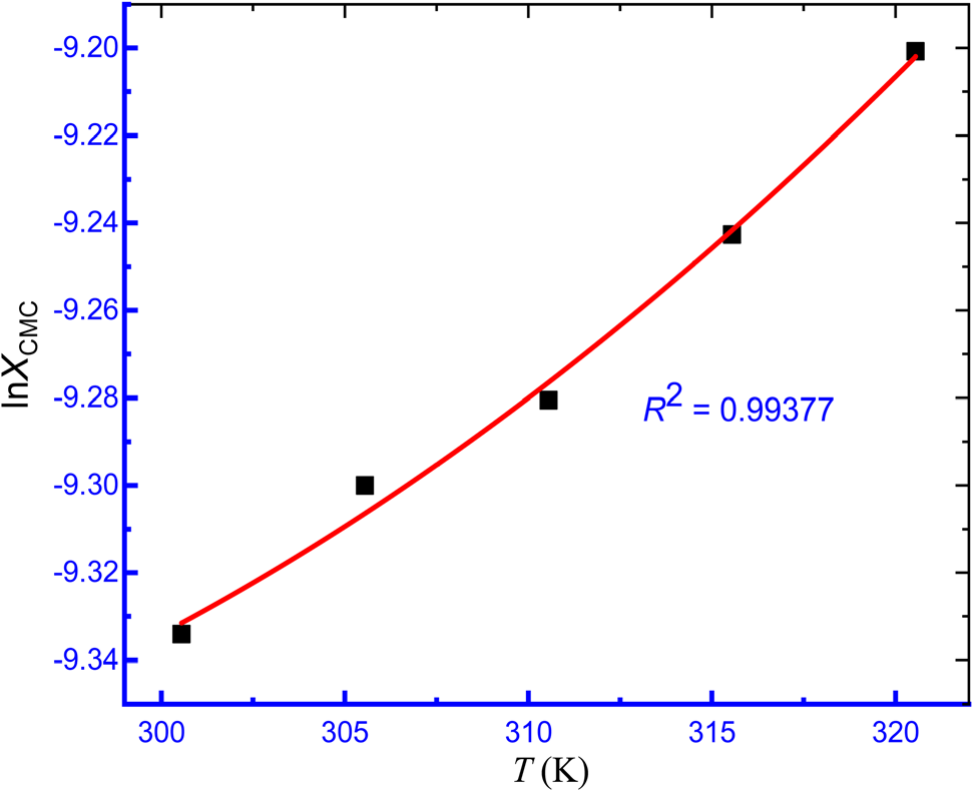
ln *X*_CMC_*versus T* plot for the assemblage of TTAB + 0.30 mmol kg^−1^ CMT mixed system in 0.50 mmol kg^−1^ aq. NaBz solution.

The regressive illustration of least squares can be used to determine the constants *A*, *B*, and *C* in the last equation (eqn (3)). The plot of ln *X*_CMC_*vs.* temperature of micellization of TTAB + CMT mixture in aqueous hydrotropic solution is presented in Fig. 5, and this second-order polynomial fitting curve has been used to determine the values of standard enthalpy change (Δ*H*^0^_m_) of the aggregation process. Table 3 lists the constant values (*A*, *B*, and *C*) for eqn (3). Consequently, the following equation (eqn (4)) was applied to compute the Δ*H*^0^_m_ values of the association of surfactant.

4 Δ*H*^0^_m_ = −(1 + *β*)*RT*^2^[*B* + 2*CT*]

**Table 3:** Values of regressive constants (*A*, *B*, and *C*) for the TTAB + CMT mixed system to obtain the standard enthalpy changes (Δ*H*^0^_m_)

Medium	*C*_HTs_ (mmol kg^−1^)	*A*	*B*	*C*
H_2_O + NaBz	0.500	−1.9098	−0.0539	0.0001
H_2_O + NaS	0.500	−41.034	0.194	−0.0003
H_2_O + ReSC	10.00	12.967	−0.1566	0.0003
H_2_O + PABA	10.00	−4.2268	−0.0435	0.00009

Finally, by using the following equation, the values of the standard entropy change (Δ*S*^0^_m_), for the development of micelle of surfactant mixture, were obtained.^78–80^

5 Δ*S*^0^_m_ = (Δ*H*^0^_m_ − Δ*G*^0^_m_)/*T*

**Fig. 6:**
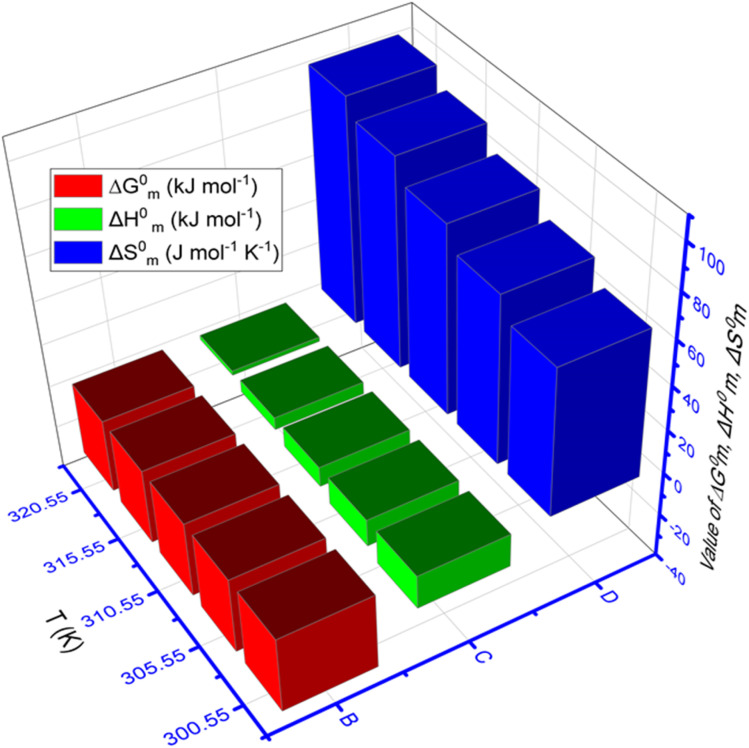
Three-dimensional plot of temperature *vs.* thermodynamic parameters (Δ*G*^0^_m_, Δ*H*^0^_m_, and Δ*S*^0^_m_) of TTAB + CMT mixed system in the attendance of 0.50 mmol kg^−1^ NaS at several experimental temperatures.

The values of Δ*G*^0^_m_, Δ*H*^0^_m_, and Δ*S*^0^_m_ for TTAB + CMT mixture containing 0.30 mmol kg^−1^ CMT in H_2_O + HTs medium, obtained at various temperatures, are shown in Table 4. Also, Fig. 6 shows the three-dimensional plot of temperature *vs.* thermodynamic parameters (Δ*G*^0^_m_, Δ*H*^0^_m_, and Δ*S*^0^_m_) of TTAB + CMT mixed system in the appearance of NaS (0.50 mmol kg^−1^ in each case) at working temperatures. To define the interactions between the drug and surfactants, the parameters related to thermodynamics, such as Δ*G*^0^_m_, Δ*H*^0^_m_, and Δ*S*^0^_m_ play a vital role in this current study. The values of Δ*G*^0^_m_ for all studied system were found negative that demonstrates all the experiments under investigation were spontaneous. The −Δ*G*^0^_m_ values for cationic surfactant in presence of polymer was also reported in the literature.^35^ The negative values of Δ*G*^0^_m_ decreased with the enhancement of temperature for IHTs, which indicates that the process of micellization was less spontaneous at higher temperatures resembling the upward trend of CMC value,^81^ while the values of Δ*G*^0^_m_ were almost constant in the introduction of NHTs (Table 4). In the literature, both upward and downward trends for −Δ*G*^0^_m_ value with the change of thermal condition of experiment for the aggregation process also reported.^57,58,77,78^

**Table 4:** The values of Δ*G*^0^_m_, Δ*H*^0^_m_, and Δ*S*^0^_m_ for TTAB + 0.30 mmol kg^−1^ CMT mixture in H_2_O + HTs solution at different temperatures^a^

Medium	*C*_HTs_ (mmol kg^−1^)	*T* (K)	Δ*G*^0^_m_ (kJ mol^−1^)	Δ*H*^0^_m_ (kJ mol^−1^)	Δ*S*^0^_m_ (J mol^−1^ K^−1^)	Δ*C*^0^_m_ (kJ mol^−1^ K^−1^)
H_2_O + NaBz	0.500	300.55	−38.25	−7.649	101.8	−0.223
		305.55	−37.80	−8.954	94.41	
		310.55	−37.86	−10.40	88.42	
		315.55	−34.92	−10.98	75.86	
		320.55	−34.33	−12.21	69.00	
H_2_O + NaS	0.500	300.55	−35.35	−15.40	66.39	0.671
		305.55	−35.35	−12.34	75.32	
		310.55	−35.28	−9.040	84.50	
		315.55	−35.21	−5.606	93.82	
		320.55	−34.38	−1.997	101.0	
H_2_O + ReSC	10.00	300.55	−40.67	−30.12	35.11	−0.965
		305.55	−40.91	−34.86	19.80	
		310.55	−40.64	−39.33	4.20	
		315.55	−40.78	−44.44	−11.58	
		320.55	−40.55	−49.45	−27.75	
H_2_O + PABA	10.00	300.55	−39.78	−13.37	87.86	−0.316
		305.55	−39.80	−14.82	81.75	
		310.55	−40.00	−16.40	75.97	
		315.55	−40.11	−18.06	69.88	
		320.55	−40.05	−19.65	63.64	

a Standard deviation: Δ*G*^0^_m_ = 4%, Δ*H*^0^_m_ = 5%, Δ*S*^0^_m_ = 6%, and Δ*C*^0^_m_ = 5%.

The values of Δ*H*^0^_m_ and Δ*S*^0^_m_ are the key factors in determining the supramolecular interactions between drug (CMT) and surfactant (TTAB) in the presence of HTs. In the present study, the values of Δ*H*^0^_m_ for every experimental trial were found negative manifesting the process was exothermic reactions (Table 4). The order of negative Δ*H*^0^_m_ values for the different medium at 310.55 K is; −Δ*H*^0^_m_ (ReSC) > −Δ*H*^0^_m_ (PABA) > −Δ*H*^0^_m_ (NaBz) > −Δ*H*^0^_m_ (NaS). The negative values of Δ*H*^0^_m_ experienced to be augmented with the boost of temperature in NaBz, ReSC, and PABA media, which reveals that the micelle creation scheme is more exothermic at higher working temperatures in these media. This value followed an opposite trend in the NaS medium, while less exothermic in NaS medium. The next parameter, standard entropy change had positive values for all experimental sets of investigation, except the one observed in the aqueous solution of ReSC (315.55 K and 320.55 K) having a negative value.

**Fig. 7:**
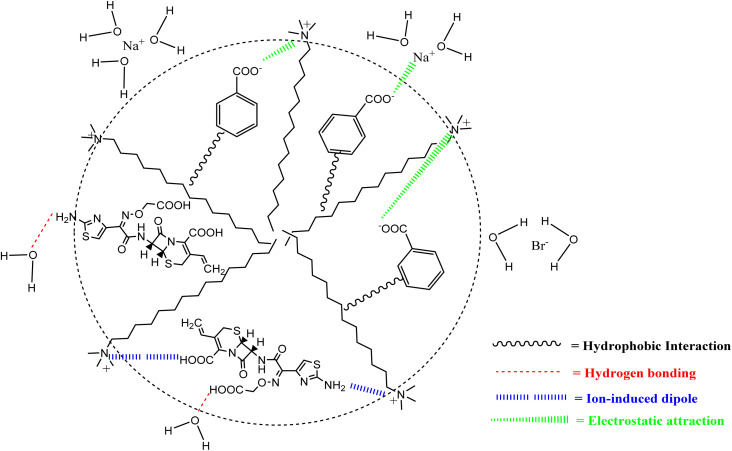
A schematic diagram demonstrating the plausible interaction forces between TTAB and CMT drug in the presence of NaBz.

The Δ*S*^0^_m_ value was found a downward trend for aqueous solution of NaBz, ReSC, and PABA, while NaS showed an upward trend with increasing temperature (Table 4). The Δ*S*^0^_m_ values that are positive signify a rise in chaos, disturbance, or entropy inside a system. They are linked to phenomena, like heat absorption, phase shifts, and spontaneous processes that cause more systemic disorder. Naturally, a positive Δ*S*^0^_m_ is associated with spontaneity. The spontaneous processes tend to occur without an external intervention because they lead to a higher entropy state, which is favored. Consequently, the process of aggregation of TTAB + CMT mixtures was entropically favored and spontaneous, which supports the findings of information obtained by −Δ*G*^0^_m_ in this investigation. The observed positive Δ*S*^0^_m_ values for TTAB + CMT accumulation may be explained by two issues. The first one involves the demolishing of iceberg configurations by moving the hydrated hydrophobic portion into the micellar core^37^ of a TTAB + CMT combination. Another factor is that, in contrast to the aqueous surroundings, the degree of rotational flexibility of hydrophobic parts rises in the micellar interior.^82,83^ Furthermore, the Δ*H*^0^_m_ and Δ*S*^0^_m_ values jointly reveal that the micellization of TTAB + CMT is controlled by both enthalpy and entropy in all cases, except in ReSC medium at 315.55 and 320.55 K. To find out the interaction between TTAB and CMT, both magnitude and sign of Δ*H*^0^_m_ and Δ*S*^0^_m_ have been taken under consideration. Additionally, the researcher discloses that exothermic (ion–dipole) interactions, as well as hydrophobic forces, are likely to be responsible for the binding between drug and surfactant for negative Δ*H*^0^_m_ and positive Δ*S*^0^_m_ values.^84^ The hydrophobic and exothermic (ion–dipole) interactions are likely to be responsible for the binding force between CMT and TTAB, according to the negative Δ*H*^0^_m_ and positive Δ*S*^0^_m_ values in the presence of NaBz, NaS, ReSC (except 315.55 K and 320.55 K), and PABA. But the coexistence of electrostatic and hydrophobic interaction is denoted by the −Δ*H*^0^_m_ and −Δ*S*^0^_m_ values.^85^ This type of result was found for aqueous ReSC medium at 315.55 and 320.55 K, hence, there was an electrostatic and hydrophobic interaction between TTAB and CMT. A schematic diagram is given in Fig. 7 where the plausible interaction forces between TTAB and CMT drug is demonstrated in the presence of HT.

However, at various temperatures, Rehman *et al.*^36^ discovered electrostatic interactions in the association process of ionic surfactants (CTAB and SDS) in polylactic acid (PLA), whereas Hanif *et al.*^39^ reported benzothiophene (BZT) partitioning in micellar medium, which was aided by both electrostatic and hydrophobic factors. Rouf *et al.*^35^ performed an extensive physicochemical analysis of cationic surfactant and uncharged polyethylene oxide (PEO) mixture, where they described the existence of strong interactions between the components.

### 3.5. Molar heat capacity (Δ*C*^0^_m_)

**Fig. 8:**
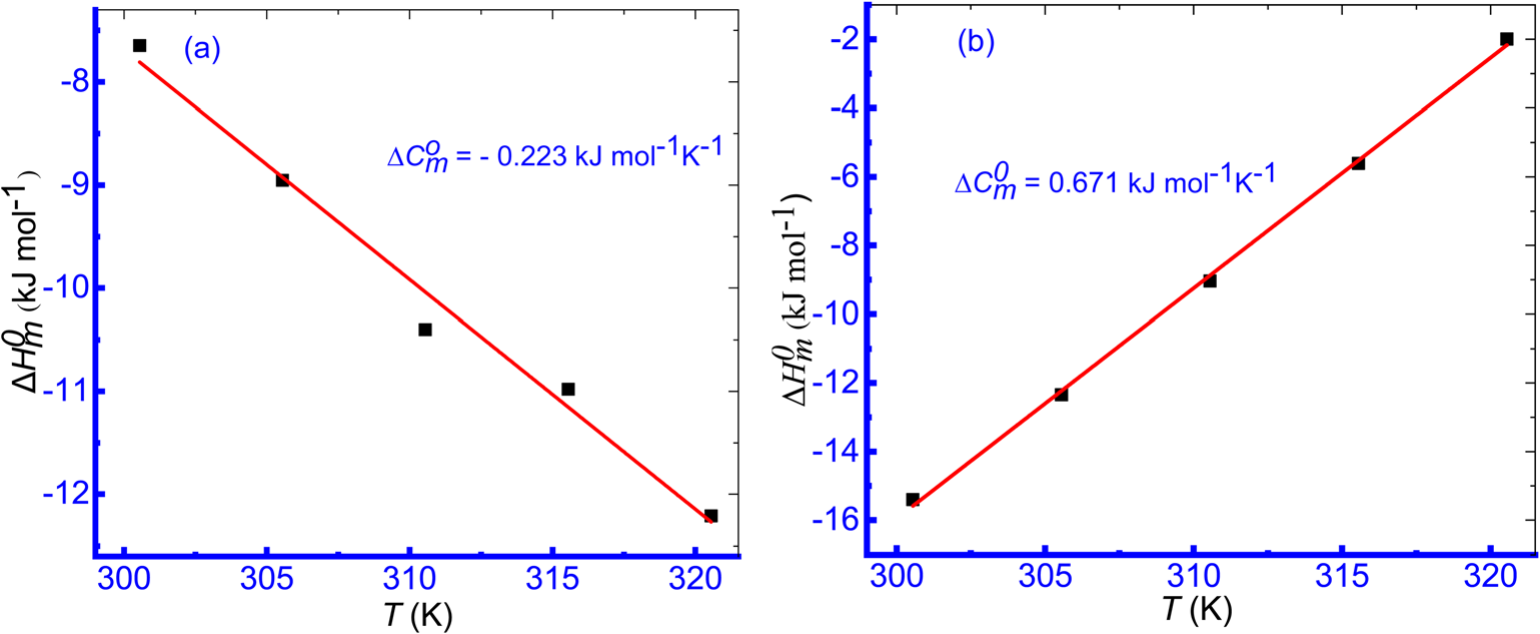
Plot of Δ*H*^0^_m_*vs. T* (K) for the TTAB + CMT (0.30 mmol kg^−1^) system in aq. 0.50 mmol kg^−1^ solutions of (a) NaBz, and (b) NaS.

Another important thermodynamic parameter is the molar heat capacity (Δ*C*^0^_m_) to represent the micellar representation of surfactant, as well as the binding characteristics of biological molecules, like protein/drug with surfactant, which is the slope value of the plot of Δ*H*^0^_m_*vs. T* (Fig. 8). The eqn (6) is employed to assess the values of molar heat capacity (Δ*C*^0^_m_).^86,87^6
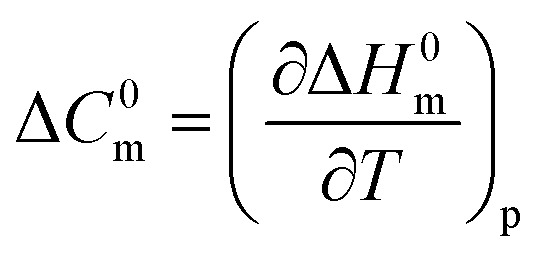


Table 4 summarizes the variations in the values of Δ*C*^0^_m_ for the micellization of TTAB + CMT in aq. hydrotropic solutions. The activities and functions of macromolecules, like proteins may be studied using the Δ*C*^0^_m_ values of micellization. This makes it useful for improving our understanding of physicochemical characteristics. For micellization of TTAB + CMT mixture, the values of Δ*C*^0^_m_ were negative in aqueous NaBz, ReSC, and PABA media. The negative values of Δ*C*^0^_m_ of micellization of TTAB + CMT mixture was obtained in aq. NaBz solution, while a positive value was found in aqueous NaS solution. The variations in Δ*C*^0^_m_ values demonstrate the reorganization of the surfactant micellar structure in the CMT drug + additives media.

### 3.6. Enthalpy–entropy compensation of micellization

**Fig. 9:**
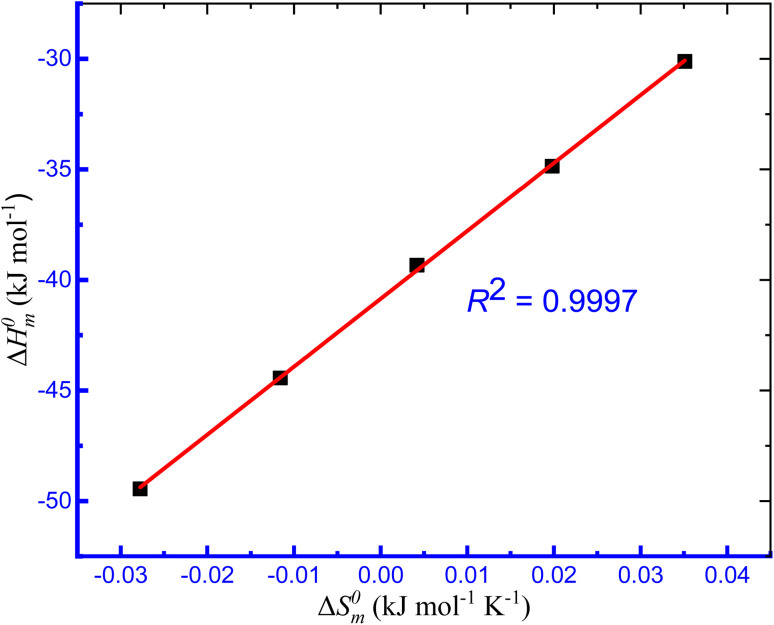
Plot of Δ*H*^0^_m_*vs.* Δ*S*^0^_m_ for the TTAB + CMT (0.30 mmol kg^−1^) mixed system in 10.00 mmol kg^−1^ solution of ReSC at different temperatures.

The values of Δ*H*^0^_m_ and Δ*S*^0^_m_ combinedly contribute to the free energy change of the micellization process of a system. The entropy and enthalpy both either control the change of free energy change or act oppositely to each other to contribute to the free energy change of the micellization of surfactant. Consequently, these two parameters compensate with each other and the compensation between the enthalpy and entropy of micellization was studied using the estimated values of Δ*H*^0^_m_ and Δ*S*^0^_m_ obtained in the CMT + TTAB mixtures in HTs solutions. The Δ*H*^0^_m_*vs.* Δ*S*^0^_m_ plot of the aggregation of TTAB + CMT mixture was found a straight line with plausible *R*^2^ values (Fig. 9) indicating that there was an excellent linear relationship between Δ*H*^0^_m_ and Δ*S*^0^_m_; and the enthalpy–entropy compensation parameters, such as the intrinsic enthalpy gain 
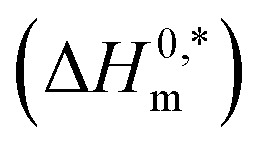
 and compensation temperature (*T*_c_) were evaluated in the studied medium. The given eqn (7) was employed to compute the enthalpy–entropy compensation parameters in the current investigation.^88–92^7
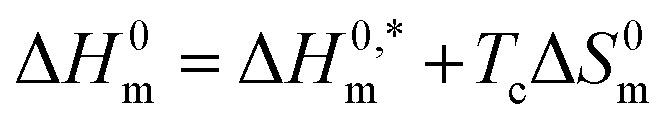


The resulting enthalpy–entropy compensation parameters in the CMT + TTAB combination were shown to substantially correlate with one another (Table 5). The compensation between enthalpy and entropy for the CMT + TTAB mixed system in the investigated HT solutions is well defined in the current work.

**Table 5:** The 
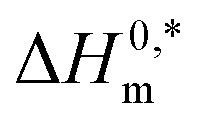
 and *T*_c_ values for the TTAB + CMT mixture in aqueous hydrotropic solutions^a^

Medium	*C*_salt_ (mmol kg^−1^)	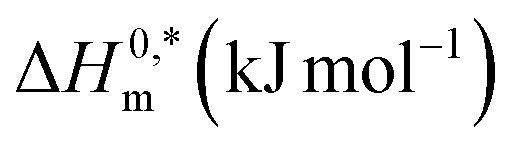	*T*_c_ (K)	*R* ^2^
H_2_O + NaBz	0.500	−21.01	128.8	0.9437
H_2_O + NaS	0.500	−40.98	381.2	0.9953
H_2_O + ReSC	10.00	−40.85	307.1	0.9997
H_2_O + PABA	10.00	−36.32	261.9	0.9994

a Standard deviation: 
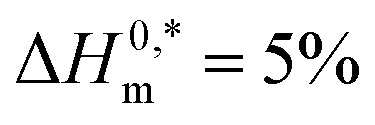
.

Despite the fact that the usual entropy change is still zero, the existence of HTs encourages the micellization of TTAB + CMT mixture due to the more negative values of Δ*H*^0^_m_. The micellar stability mostly depends on the magnitude and sign of 
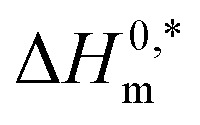
, the more negative values of 
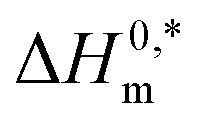
, the higher will be the stability of micelle^93^ and, again, 
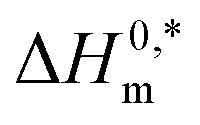
 values also monitor the solute–solute interactions.^94,95^ The micelle of TTAB + CMT in the presence of NaS and ReSC has greater stability than other solvents as the value of 
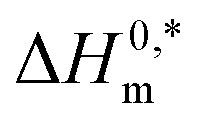
 in the presence of NaS and ReSC is more negative. The value of compensation temperature (*T*_c_) was obtained in the range of 128.8–381.2 K in the presences of both IHT and NHT. The *R*^2^ value was found in the range of 0.9437–0.9997, which represents the excellent relationship between Δ*H*^0^_m_ and Δ*S*^0^_m_ in the present study.

## 4. Conclusions

Here in the current analysis, the HTs and temperature exhibited a significant effect on the molecular association of TTAB + CMT mixture, which was examined to look for the interactions between CMT and TTAB by the conductivity measurement method. The micellar parameters (CMC and *β*) and conductivities have been altered together with the increase of temperature of the experiment and the concentration of additives. The IHTs and NHTs altered the aggregation process of TTAB + CMT mixed system differently. The CMC values initially decreased, followed by an increase with increasing concentration of NaBz and NaS, while CMC values gradually increased with the increase in concentration of PABA, indicating higher concentration of PABA created a discouraging environment for the micellization of TTAB + CMT mixture. The presence of ReSC, at lower concentration range, accelerates the formation of micelle as the CMC experienced decreasing trend with the increase of concentration in this range, although higher concentration of ReSC resulted in an increasing trend of CMC value, causing a disturbance in the formation of micelles. Among the additives medium, lower concentration of PABA caused a minimum CMC value, expressing a relatively better micellization environment for the TTAB + CMT mixture. The thermal agitation and altered hydration of the studied system, at higher temperature, disfavoured the micellization of TTAB + CMT mixture. The micellization of TTAB + CMT was spontaneous in all HTs media, observed in all temperature ranges studied, as the Δ*G*^0^_m_ values were negative. The spontaneity of micellization experienced a reduction with the boost of temperature, in almost all of the cases thoroughly investigated. The reduced counterion binding at an elevated temperature might be responsible for less spontaneity of micellization. The negative values of Δ*H*^0^_m_ and positive values of Δ*S*^0^_m_ clearly notify that exothermic (ion–dipole) interactions, as well as hydrophobic interactions are the key contributing forces between the drug, CMT, and TTAB surfactant. Furthermore, the −Δ*H*^0^_m_ and +Δ*S*^0^_m_ values jointly demonstrate that the micellization of TTAB + CMT is controlled by both enthalpy and entropy in all cases, except the one observed in ReSC medium at 315.55 and 320.55 K. The 
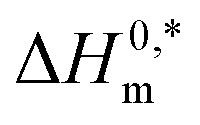
 values reveal that the highest stability of TTAB + CMT is in NaS medium, while the lowest is in NaBz medium. The value of compensation temperature (*T*_c_) was obtained 128.8–381.2 K range in the presences of both IHTs and NHTs. With a few minor exceptions, the *T*_c_ values of TTAB + CMT mixture in the used solvents showed a behavior that was very similar to that of the values observed in biological fluids. The mode of interaction among the studied drug and surfactant, as well as the stability of the micelle will help in order to formulate drug in a better and most successful way.

## Conflicts of interest

There are no conflicts of interest to declare.

## Supplementary Material

## References

[cit1] Shaban S. M., Kim D. H. (2020). Korean J. Chem. Eng..

[cit2] Naqvi A. Z., Noori S., Kabir-ud-Din (2016). RSC Adv..

[cit3] Bagheri A., Khalili P. (2017). RSC Adv..

[cit4] Chakraborty T., Chakraborty I., Ghosh S. (2011). Arabian J. Chem..

[cit5] Lawrence M. J. (1994). Chem. Soc. Rev..

[cit6] Drummond C. J., Fong C. (1999). Curr. Opin. Colloid Interface Sci..

[cit7] Torchilin V. P. (2001). J. Controlled Release.

[cit8] Chen J., Dickinson E. (1995). Colloids Surf., A.

[cit9] Otzen D. (2011). Biochim. Biophys. Acta, Proteins Proteomics.

[cit10] Singh P., Cameotra S. S. (2004). Trends Biotechnol..

[cit11] Goddard E. D. , and Ananthapadmanabhan K. P., Interactions of Surfactants with Polymers and Proteins, 1993

[cit12] Tam K. C., Wyn-Jones E. (2006). Chem. Soc. Rev..

[cit13] Taylor D. J. F., Thomas R. K., Penfold J. (2007). Adv. Colloid Interface Sci..

[cit14] Talele P., Kishore N. (2014). J. Chem. Thermodyn..

[cit15] Javed M., Iqbal S., Fatima I., Nadeem S., Mohyuddin A., Arif M., Amjad A., Shahid S., Alshammari F. H., Alahmdi M. I., Elkaeed E. B., Alzhrani R. M., Awwad N. S., Ibrahium H. A., Qayyum M. A. (2022). Colloid Interface Sci. Commun..

[cit16] Hoque M. A., Patoary M. O. F., Molla M. R., Halim M. A., Khan M. A., Rub M. A. (2017). J. Dispersion Sci. Technol..

[cit17] Mahbub S., Akter S., Luthfunnessa, Akter P., Hoque M. A., Rub M. A., Kumar D., Alghamdi Y. G., Asiri A. M., Džudžević-Čančar H. (2020). RSC Adv..

[cit18] Sergeeva V. F. (1965). Russ. Chem. Rev..

[cit19] Roy B. K., Moulik S. P. (2003). Curr. Sci..

[cit20] Hodgdon T. K., Kaler E. W. (2007). Curr. Opin. Colloid Interface Sci..

[cit21] Valeeva F. G., Kuryashov D. A., Zakharov S. V., Vagapova G. I., Vasilieva E. A., Bashkirtseva N. Y., Zakharova L. Y., Konovalov A. I. (2013). Russ. Chem. Bull..

[cit22] Vasilieva E. A., Zakharov S. V., Kuryashov D. A., Valeeva F. G., Ibragimova A. R., Bashkirtseva N. Y., Zakharova L. Y. (2015). Russ. Chem. Bull..

[cit23] Valeeva F. G., Vasilieva E. A., Gaynanova G. A., Kashapov R. R., Zakharov S. V., Kuryashov D. A., Lukashenko S. S., Bashkirtseva N. Y., Zakharova L. Y. (2015). J. Mol. Liq..

[cit24] Dey A., Sandre V., Marangoni D. G., Ghosh S. (2018). J. Phys. Chem. B.

[cit25] Neuberg C. (1916). Biochem.
Z..

[cit26] Naqvi A. Z., Rub M. A., Kabir-ud-Din (2021). J. Colloid Interface Sci..

[cit27] Schreinemachers P., Simmons E. B., Wopereis M. C. S. (2018). Global Food Secur..

[cit28] Chipley J. R. , Antimicrobials in Food, CRC Press, Florida, 3rd edn, 2005, pp. 11–48

[cit29] Amann R., Peskar B. A. (2002). Eur. J. Pharmacol..

[cit30] Kluczyk A., Popek T., Kiyota T., de Macedo P., Stefanowicz P., Lazar C., Konishi Y. (2002). Curr. Med. Chem..

[cit31] Schmiedel K. W. and Decker D., Resorcinol. Ullmann's Encyclopedia of Industrial Chemistry, Wiley-VCH, Weinheim, 2012

[cit32] Boer J., Jemec G. B. E. (2010). Clin. Exp. Dermatol..

[cit33] Wipperman J., Bragg D. A., Litzner B. (2019). Am. Fam. Physician.

[cit34] Rehman N., Irshad Ul Haq M., Ullah H., Sadiq M., Khan A., Mian I. U. (2023). Z. Phys. Chem..

[cit35] Rauf A., Baloch M. K., Khan A., Khan Z., Rauf S. (2016). J. Chil. Chem. Soc..

[cit36] Rehman N., Khan A., Bibi I., Siddiq M. (2012). Chin. J. Polym. Sci..

[cit37] Khan A., Siddiq M. (2016). J. Dispersion Sci. Technol..

[cit38] Ali H., Khan A., Ahmad T., Dervisi A., Fallis I., Shoetan I. O., Khan A., Hussain A. (2022). J. Surfactants Deterg..

[cit39] Hanif S., Usman M., Hussain A., Rasool N., Khan A., Jamal M. A., Elgorban A. M., Rana U. A. (2016). J. Surfactants Deterg..

[cit40] Sachin K. M., Karpe S. A., Singh M., Bhattara A. (2019). R. Soc. Open Sci..

[cit41] Alfaifi S. Y. M., Kumar D., Rub M. A., Khan F., Azum N., Khan A., Asiri A. M., Čančar H. D. (2021). Korean J. Chem. Eng..

[cit42] Islam M. N., Rub M. A., Islam M. R., Goni M. A., Rana S., Kumar D., Asiri A. M., Alghamdi Y. G., Hoque M. A., Kabir S. E. (2023). J. Mol. Liq..

[cit43] Rajput S. M., Mondal K., Kuddushi M., Jain M., Ray D., Aswal V. K., Malek N. I. (2020). Colloid Interface Sci. Commun..

[cit44] Rub M. A., Hasan T., Akter R., Kumar D., Kabir-ud-Din, Asiri A. M., Hoque M. A. (2023). J. Mol. Liq..

[cit45] Paul R., Chattaraj K. G., Paul S. (2021). Langmuir.

[cit46] Rub M. A., Asiri A. M., Azum N., Kabir-ud-Din (2014). J. Ind. Eng. Chem..

[cit47] Kumar D., Khan F., Rub M. A., Azum N., Asiri A. M. (2021). J. Mol. Liq..

[cit48] Khan A., Khan S., Khan N., Naz S., Bououdina M., Rehman N., Humayun M., Shah N., Anwar N., Ali H. (2023). Z. Phys. Chem..

[cit49] Gorski N., Gradzielski M., Hoffmann H. (1994). Langmuir.

[cit50] Cocke D. L., Schennach R., Yu Z. (2002). J. Chromatogr. Sci..

[cit51] Grayson M. L. , Kucers' The Use of Antibiotics: A Clinical Review of Antibacterial, Antifungal, Antiparasitic and Antiviral Drugs, Boca Raton, 7th edn, 2017

[cit52] Ahmed F., Molla M. R., Saha M., Shahriar I., Rahman M. S., Halim M. A., Rub M. A., Hoque M. A., Asiri A. M. (2019). RSC Adv..

[cit53] Rub M. A., Azum N. (2021). J. Mol. Liq..

[cit54] Hasan M. Z., Mahbub S., Hoque M. A., Rub M. A., Kumar D. (2020). J. Phys. Org. Chem..

[cit55] Hoque M. A., Ali M. I., Rub M. A., Rahman M., Rana S., Rahman M. M., Kumar D., Azum N., Asiri A. M., Khan M. A. (2023). Int. J. Biol. Macromol..

[cit56] Kumar D., Rub M. A. (2020). J. Mol. Liq..

[cit57] Gonzalez-perez A., Prieto G., Ruso J. M., Sarmiento F. (2003). Mol. Phys..

[cit58] Abedin J., Mahbub S., Rahman M. M., Hoque M. A., Kumar D., Khan J. M., EI- Sherbeeny A. M. (2021). Chin. J. Chem. Eng..

[cit59] Banjare R. K., Banjare M. K., Panda S. (2020). J. Solution Chem..

[cit60] Ghosh S. (2001). J. Colloid Interface Sci..

[cit61] Ali B. A., Zughul M. B., Badwan A. A. (1995). J. Dispersion Sci. Technol..

[cit62] Wu P., Liu G., Li X., Peng Z., Zhou Q., Qi T. (2020). JOM.

[cit63] Zhang Y., Li Y., Songa Y., Lia J. (2015). RSC Adv..

[cit64] Varade D., Bahadur P. (2004). J. Surfactants Deterg..

[cit65] Vasilieva E. A., Vasileva L. A., Valeeva F. G., Karimova T. R., Zakharov S. V., Lukashenko S. S., Kuryashov D. A., Gaynanova G. A., Bashkirtseva N. Y., Zakharova L. Y. (2020). Surf. Innovations.

[cit66] Akram M., Yousuf S., Sarwar T., Kabir-ud-Din (2014). Colloids Surf., A.

[cit67] Owoyomi O., Ogunlusi G. O., Olatan O. M. (2016). Phys. Chem. Liq..

[cit68] Bunton C. A., Nome F., Quina F. H., Romsted L. S. (1991). Acc. Chem. Res..

[cit69] EI-Khordagui L. K. (1982). Int. J. Pharm..

[cit70] Akbaş H., Kartal Ç. (2006). Colloid J..

[cit71] Naorem H., Devi S. D. (2006). J. Surf. Sci. Technol..

[cit72] Coetzee J. F. and Ritchie C. D., Solute–Solvent Interaction, Marcel Dekker, New York, 1969

[cit73] Harned H. S. and Owen B. B., The Physical Chemistry of Electrolyte Solutions, Reinhold, New York, 3rd edn, 1958

[cit74] Callaghan A., Doyle R., Alexander E., Palepu R. (1993). Langmuir.

[cit75] Islam M., Hossain M., Mahbub S., Hoque M. A., Kumar D., Wabaidur S. M., Habila M. A., AL-Anaz M., Kabir M. (2021). Mol. Phys..

[cit76] Rub M. A., Azum N., Khan S. B., Marwani H. M., Asiri A. M. (2015). J. Mol. Liq..

[cit77] Joy M. T. R., Hossain M. A. A., Khatun M. J., Rub M. A., Hossain M. D., Azum N., Anis-Ul-Haque K. M., Mohanta S. C., Hoque M. A., Asiri A. M., Kabir S. E. (2023). J. Mol. Liq..

[cit78] Clint J. H. , Surfactant Aggregation, Springer Science & Business Media, 2012

[cit79] Attwood D. , Surfactant Systems Their Chemistry, Pharmacy and Biology, 1983

[cit80] Rosen M. J. , and Kunjappu J. T., Surfactants and Interfacial Phenomena, John Wiley & Sons, 2012

[cit81] Amin M. R., Molla M. R., Rana S., Hoque M. A., Rub M. A., Kabir M., Asiri A. M. (2019). Phys. Chem. Liq..

[cit82] Chen L. J., Lin S. Y., Huang C. C., Chen E. M. (1998). Colloids Surf., A.

[cit83] Stainsby G., Alexander A. (1950). Trans. Faraday Soc..

[cit84] Sultana S., Rahman M. M., Amin M. R., Rana S., Hoque M. A., Kumar D., Alfakeer M. (2021). Mol. Phys..

[cit85] Reeves R. L., Kaiser R. S., Mark H. W. (1973). J. Colloid Interface Sci..

[cit86] Owoyomi O., Ige J., Soriyan O. O. (2011). Chem. Sci. J..

[cit87] Jelesarov I., Bosshard H. R. (1999). J. Mol. Recognit..

[cit88] Amin M. R., Mahbub S., Hidayathulla S., Alam M. M., Hoque M. A., Rub M. A. (2018). J. Mol. Liq..

[cit89] Sharma P., Kumar H., Singla M., Kumar V., Ghfar A. A., Pandey S. (2022). J. Mol. Liq..

[cit90] Joy M. T. R., Rub M. A., Hossai M. A. A., Biswas P. K., Alghamdi Y. G., Asiri A. M., Amin M. R., Mohanta S. C., Hoque M. A., Kabir S. E. (2022). Mol. Phys..

[cit91] Ali M. M., Hasan T., Khan J. M., Kumar D., Ahmad A., Rana S., Rahman M. M., Hoque M. A., Kabir S. E. (2023). RSC Adv..

[cit92] Ahmed M. R., Rub M. A., Ali M. I., Rana S., Rahman M., Kumar D., Asiri A. M., Hoque M. A. (2022). J. Mol. Liq..

[cit93] Rub M. A., Sheikh M. S., Khan F., Azum N., Alghamdi Y. G., Asiri A. M. (2021). J. Mol. Liq..

[cit94] Medoš Ž., Bester-Rogac M. (2015). J. Chem. Thermodyn..

[cit95] Hasan T., Mahbub S., Kumar D., Gatasheh M. K., Joy M. T. R., Goni M. A., Rana S., Hoque M. A. (2022). Mol. Phys..

